# Supplementary material for: Downregulation of IL-8 and IL-10 by LRRC8A Inhibition through the NOX2–Nrf2–CEBPB Transcriptional Axis in THP-1-Derived M2 Macrophages
Source: Int J Mol Sci. 2024 Sep 5;25(17):9612. doi: 10.3390/ijms25179612 (PMC11395230; doi:10.3390/ijms25179612)
Supplement: Supplementary file 1 [file ijms-25-09612-s001.zip › ijms-3171944-supplementary.pdf]

Supplementary Materials

Down-regulation of IL-8 and IL-10 by LRRC8A inhibition through the NOX2-Nrf2-CEBPB transcriptional axis in THP-1-derived M_2 macrophages

Miki Matsui [1,#], Junko Kajikuri [1,#], Hiroaki Kito [1], Elghareeb E. Elboray [2,3], Takayoshi Suzuki [2], Susumu Ohya [1,*]

[1]Department of Pharmacology, Graduate School of Medical Sciences, Nagoya City University, Nagoya 467-8601, Japan; c241739@ed.nagoya-cu.ac.jp (M.M.); kajikuri@med.nagoya-cu.ac.jp (J.K.); kito@med.nagoya-cu.ac.jp (H.K.); sohya@med.nagoya-cu.ac.jp (S.O.)
[2]Department of Complex Molecular Chemistry, SANKEN, Osaka University, Osaka, Japan; tkyssuzuki@sanken.osaka-u.ac.jp (T.S.).
[3] Department of Chemistry, Faculty of Science, South Valley University, Qena, Egypt; e.elboray@sci.svu.edu.eg (E.E.E.)

Supplementary Materials: The following are available online at www.mdpi.com/xxx/s1

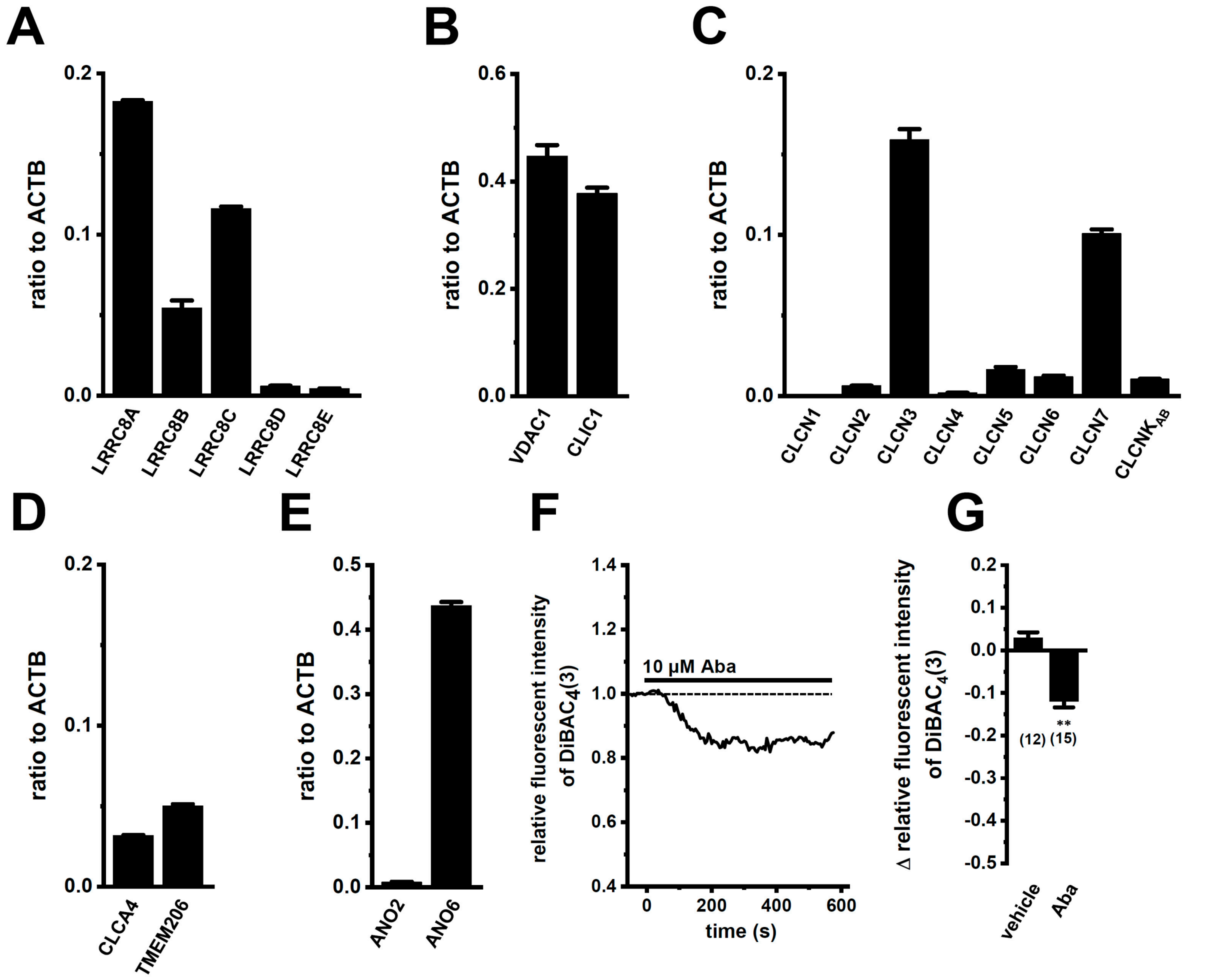

Figure S1. Molecular identification of LRRC8, VDAC, CLIC, CLCN, and ANO isoforms and effects of the ANO6 inhibitor, abamectin on membrane potential in M_2-MACs. A-E: Real-time PCR examination of LRRC8s (A), VDAC1/CLIC1 (B), CLCNs (C), CLCA4/TMEM206 (D), and ANO2/6 (E) in M_2-MACs (*n* = 4 for each). Expression levels are shown as a ratio to ACTB. F: Measurement of changes in membrane potential following the application of the ANO6 inhibitor, abamectin (Aba, 10 μM) using the voltage-sensitive dye, $DiBAC_4(3)$. The relative time course of changes in fluorescence intensities (1.0 at time 0 s) in a single M_2-MAC was shown. G: Summarized results of Aba (10 μM)-induced hyperpolarizing responses. Numbers used for experiments are shown in parentheses. **: P <0.01 vs. the vehicle control.

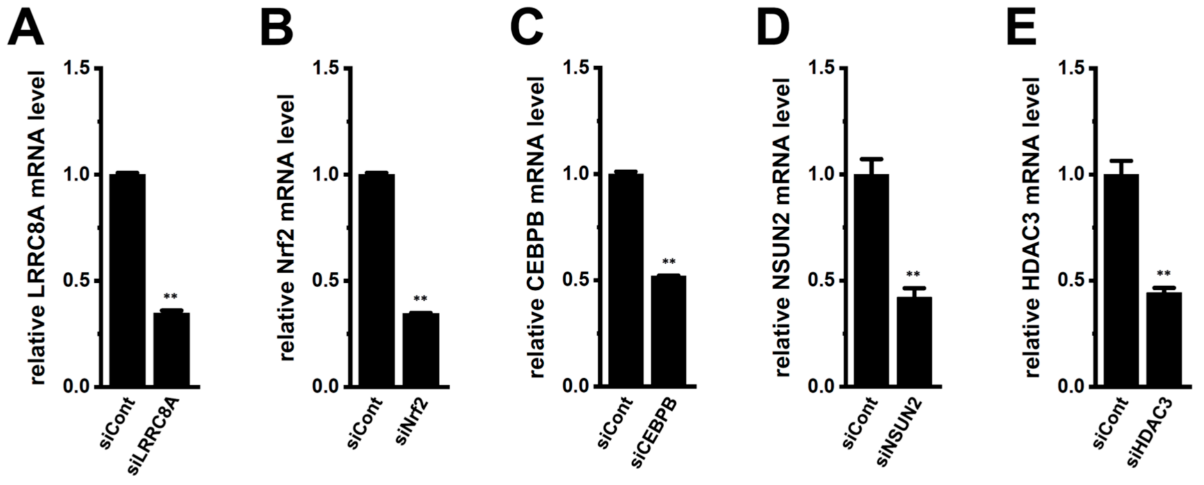

Figure S2. The transcriptional repression efficacy of siRNAs in M2-MACs. A-E: Real-time PCR examination of LRRC8A (A), Nrf2 (B), CEBPB (C), NSUN2 (D), and HDAC3 (E) in M2-MACs transfected with siCont, siLRRC8A, siNrf2, siCEBPB, siNSUN2, and siHDAC3 for 48-hr, respectively. After normalization to the ACTB mRNA level, the mRNA level in siCont is expressed as 1.0 (n = 4 for each). **: p <0.01 vs. siCont.

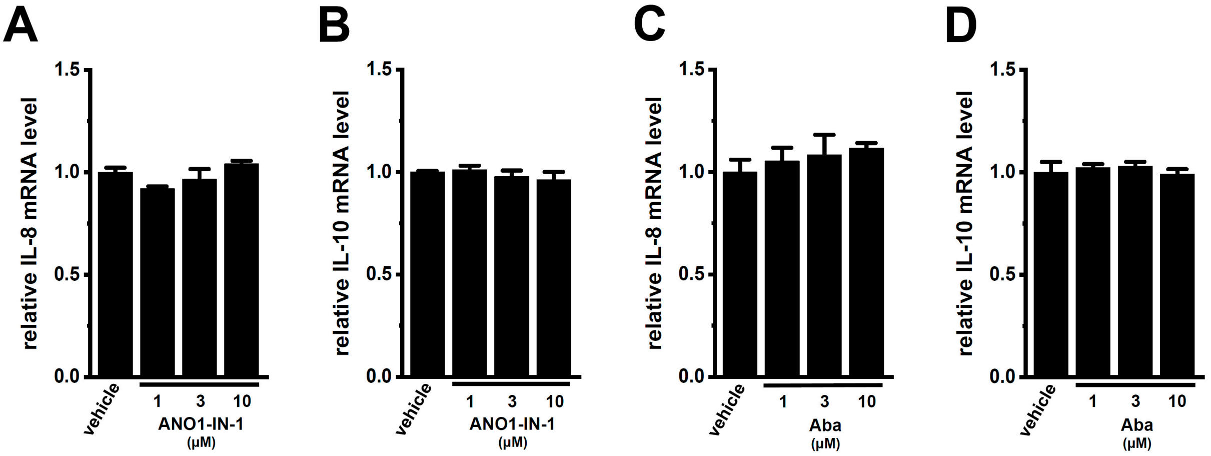

Figure S3. Effects of the pharmacological inhibition of ANO1 and ANO6 on IL-8 and IL-10 expression in M2-MACs. A-D: Real-time PCR examination of IL-8 (A, C) and IL-10 (B, D) expression in vehicle-, the ANO1 inhibitor, ANO1-IN-1 (1, 3, and 10 μM)-, and abamectin (Aba, 1, 3, and 10 μM)-treated M2-MACs for 12 hr. The mRNA expression level in the vehicle control is expressed as 1.0 (n = 4 for each).

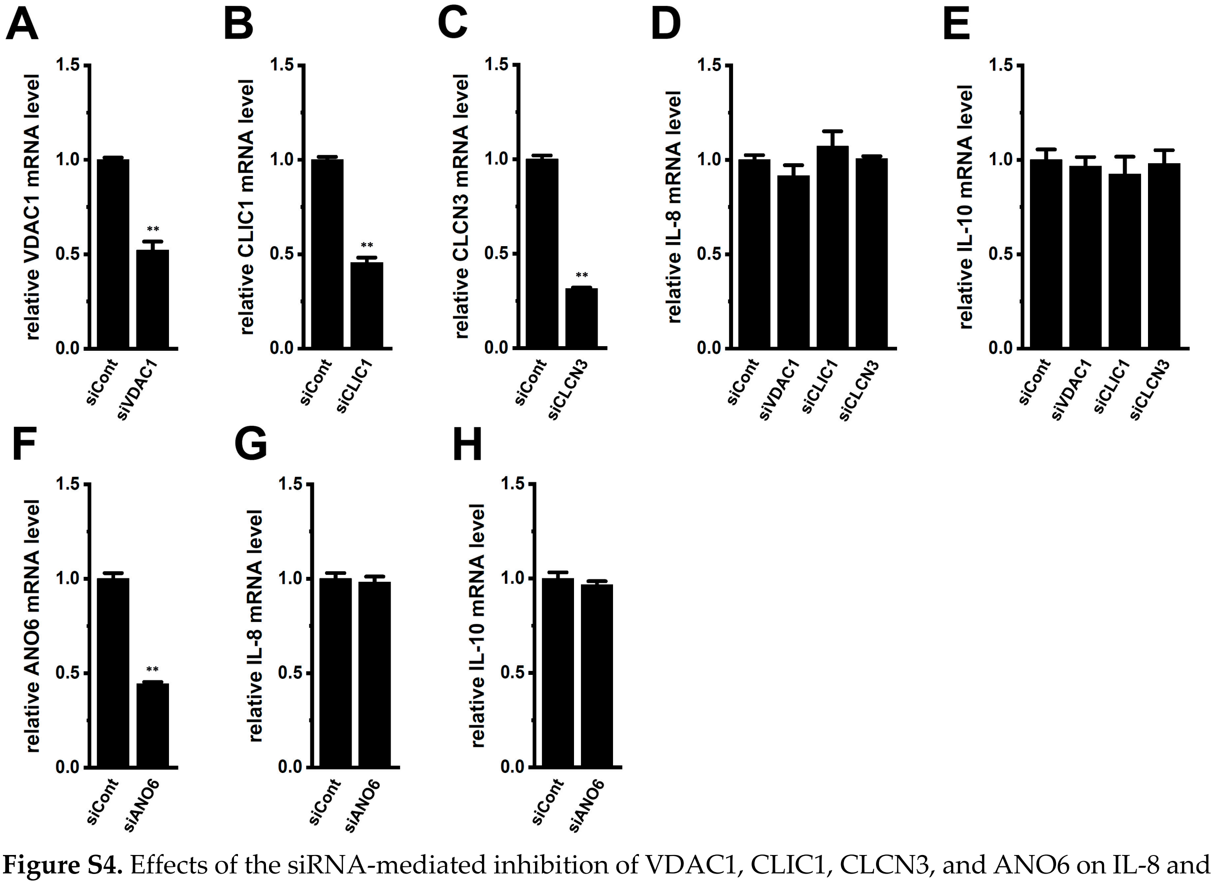

Figure S4. Effects of the siRNA-mediated inhibition of VDAC1, CLIC1, CLCN3, and ANO6 on IL-8 and IL-10 expression in M2-MACs. A, B, C, F: The transcriptional repression efficacy of siRNAs in M2-MACs. Real-time PCR examination of VDAC1 (A), CLIC1 (B), CLCN3 (C), and ANO6 (F) in M2-MACs transfected with siCont, siVDAC1, siCLIC1, siCLCN3, and siANO6 for 48 hr (n = 4 for each). After normalization to ACTB mRNA level, the mRNA level in siCont is expressed as 1.0 (n = 4 for each). **: P

< 0.01 vs. siCont. D, E, G, H: Real-time PCR examination of IL-8 (D, G) and IL-10 (E, H) expression in M_2-MACs 48 hr after the transfection of siVDAC (D, E), siCLIC1 (D, E), siCLCN3 (D, E), and siANO6 (G, H). The mRNA expression level in the siCont group is expressed as 1.0 (*n* = 4 for each).

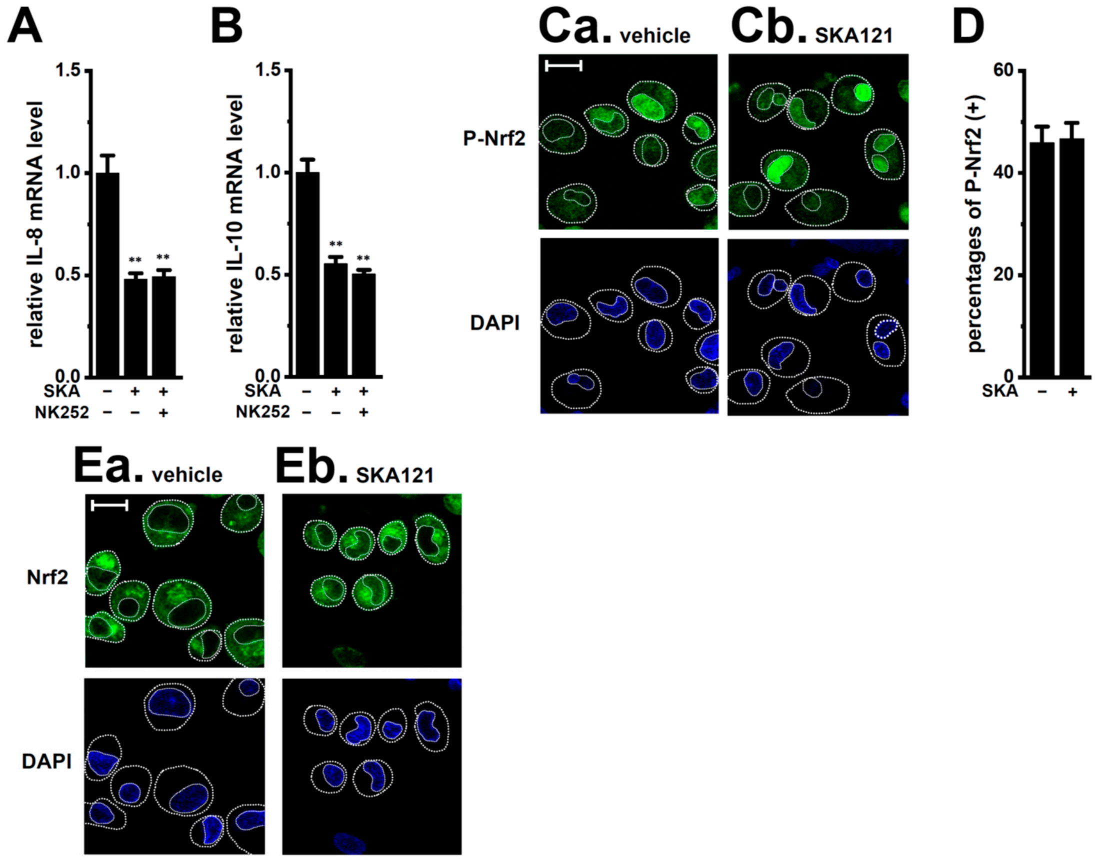

Figure S5. Effects of Nrf2 activation on the $K_{Ca}3.1$ activator, SKA-121-induced down-regulation of IL-8 and IL-10, and effects of the SKA121 treatment for 2 hr on the nuclear translocation of P-Nrf2 in M_2-MACs. A, B: Real-time PCR examination of IL-8 (A) and IL-10 (B) in M_2-MACs treated (+) or untreated (−) with 10 μM SKA121 (SKA) and 100 μM NK252 for 12 hr (*n* = 4 for each). After normalization to the ACTB mRNA level, IL-8 and IL-10 mRNA levels in the vehicle control (−/−) are expressed as 1.0. **: *P* <0.01 vs. −/−. C: Confocal fluorescent images of Alexa Fluor 488-labeled P-Nrf2 in vehicle- (Ca) and SKA121 (10 μM) (Cb)-treated M_2-MACs. D: Summarized results of the percentages of P-Nrf2-positive [P-Nrf2(+)] M_2-MACs in nuclei (*n* = 6 for each). E: Confocal fluorescent images of Alexa Fluor 488-labeled Nrf2 in vehicle- (Ea) and SKA121 (Eb)-treated M_2-MACs. Nuclear morphologies were shown by DAPI images. Thick and thin dashed lines show the plasma membrane and nuclear boundary, respectively.

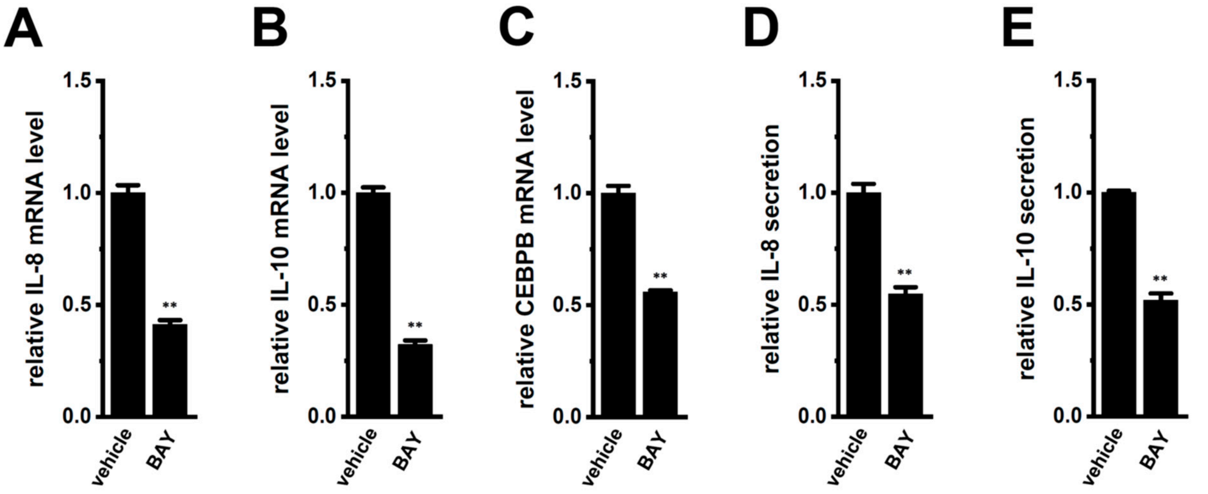

Figure S6. Effects of the pharmacological inhibition of AMPK on IL-8, IL-10, and CEPBP expression and IL-8 and IL-10 secretion in M2-MACs. A, B, C: Real-time PCR examination of IL-8 (A), IL-10 (B), and CEBPB (C) expression in vehicle- and BAY3627 (1 μM)-treated M2-MACs for 12 hr. The mRNA level in the vehicle control is expressed as 1.0 (n = 4 for each). D, E: Quantitative detection of IL-8 (D) and IL-10 (E) secretion by an ELISA assay in vehicle- and BAY3627 (1 μM)-treated M2-MACs for 24 hr. The cytokine secretion level in the vehicle control is expressed as 1.0 (n = 4 for each). **: P <0.01 vs. the vehicle control.

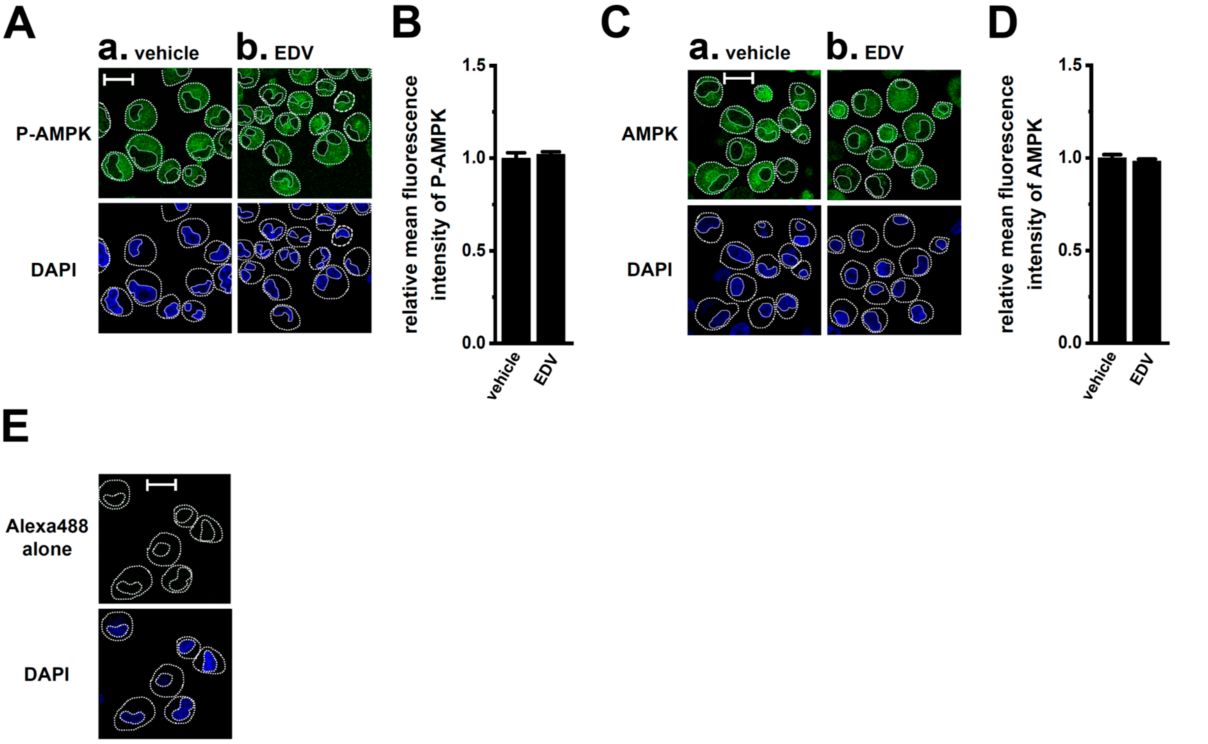

Figure S7. Effects of EDV on the cellular distribution of P-AMPK and AMPK in M2-MACs. A, C: Confocal fluorescent images of Alexa Fluor 488-labeled P-AMPK (A) and AMPK (C) in the vehicle (Aa, Ca)- and EDV (Ab, Cb)-treated M2-MACs for 2 hr. Nuclear morphologies were shown by DAPI staining. Thick and thin dashed lines show the plasma membrane and nuclear boundary, respectively. B, D: Summarized results of the relative mean fluorescence intensities of P-AMPK (B) and AMPK (D) in the cytosol regions of vehicle- and EDV-treated M2-MACs (n = 6 for each). In each batch (n = 1), more than 30 cells were observed by confocal laser scanning microscopy. E: Confocal fluorescent image of Alexa Fluor 488 alone in M2-MACs.

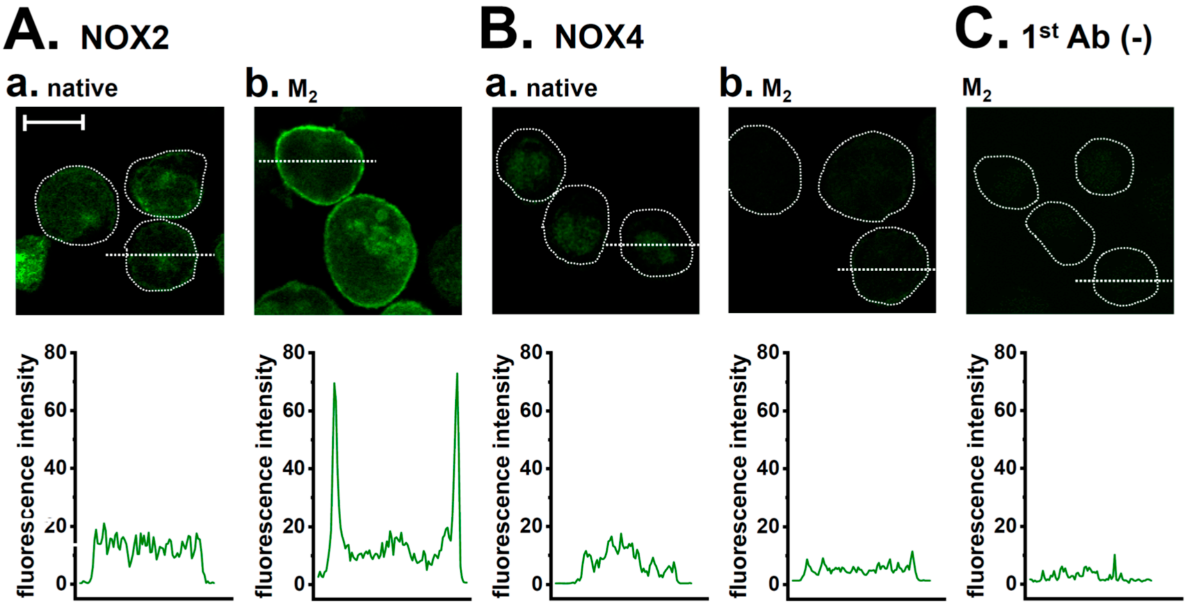

Figure S8. Immunocytochemical staining of NOX2 and NOX4 in M2-MACs. A, B: Confocal fluorescence images (green) of Alexa Fluor 488-labeled NOX2 (A) and NOX4 (B) in native THP-1 cells (Aa, Ba) and M2-MACs (Ab, Bb) (upper panels). Dashed lines show cell boundaries. Scale bars show 20 µm. Spectral line profiles of the fluorescence and quantification of fluorescent signals were performed using ImageJ software (Ver. 1.42). Fluorescence profiles on the dotted lines in Alexa Fluor 488 images are shown in green (lower panels). C: A confocal fluorescence image and the fluorescence profile of Alexa Fluor 488 alone in M2-MACs (negative control).

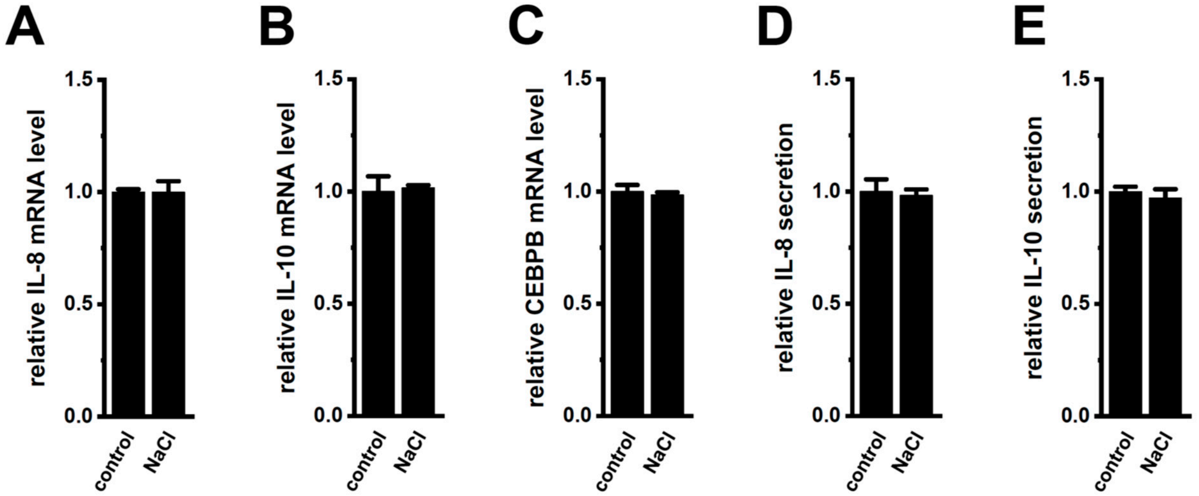

Figure S9. Effects of supplementation with 30 mM NaCl on IL-8, IL-10, and CEPBP expression and IL-8 and IL-10 secretion in M2-MACs. A, B, C: Real-time PCR examination of IL-8 (A), IL-10 (B), and CEBPB (C) expression in control- and 30 mM NaCl-supplemented M2-MACs for 24 hr. The mRNA expression level in the control is expressed as 1.0 (n = 4 for each). D, E: Quantitative detection of IL-8 (D) and IL-10 (E) secretion by an ELISA assay in both groups. The cytokine secretion level in the control is expressed as 1.0 (n = 4 for each).

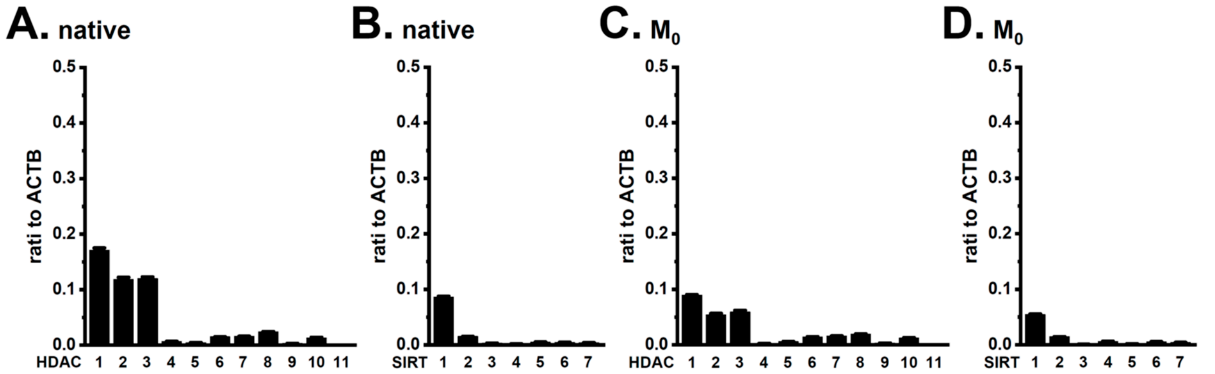

Figure S10. Identification of HDAC and SIRT isoforms expressed in native THP-1 cells and M_0-MACs. A-D: Real-time PCR examination of HDAC1-11 (A, C) and SIRT1-7 (B, D) in native THP-1 cells (A, B) and M_0-MACs (C, D). Expression levels are shown as a ratio to ACTB (n = 4 for each).

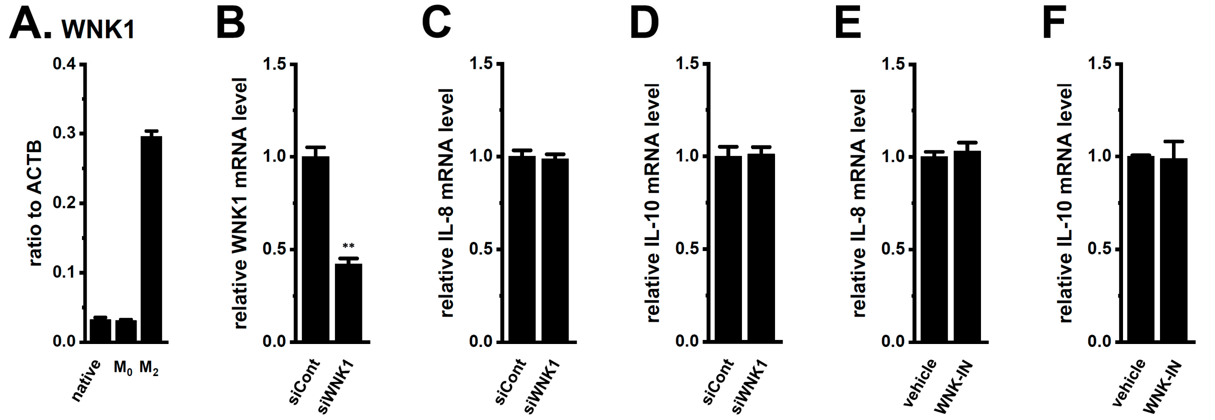

Figure S11. Effects of the pharmacological and siRNA-mediated inhibition of WNK1 on IL-8 and IL-10 expression in M_2-MACs. A: Real-time PCR examination of WNK1 expression in native THP-1 cells, M_0-MACs, and M_2-MACs. B-F: Real-time PCR examination of WNK1 (B), IL-8 (C, E), and IL-10 (D, F) expression in M_2-MACs transfected with siCont and WNK1 siRNA (siWNK1) (B-D) or treated with vehicle and WNK-IN-11 (1 μM) for 12 hr (E, F). mRNA levels in the siCont group and vehicle control are expressed as 1.0 (n = 4 for each). **: P <0.01 vs. siCont.

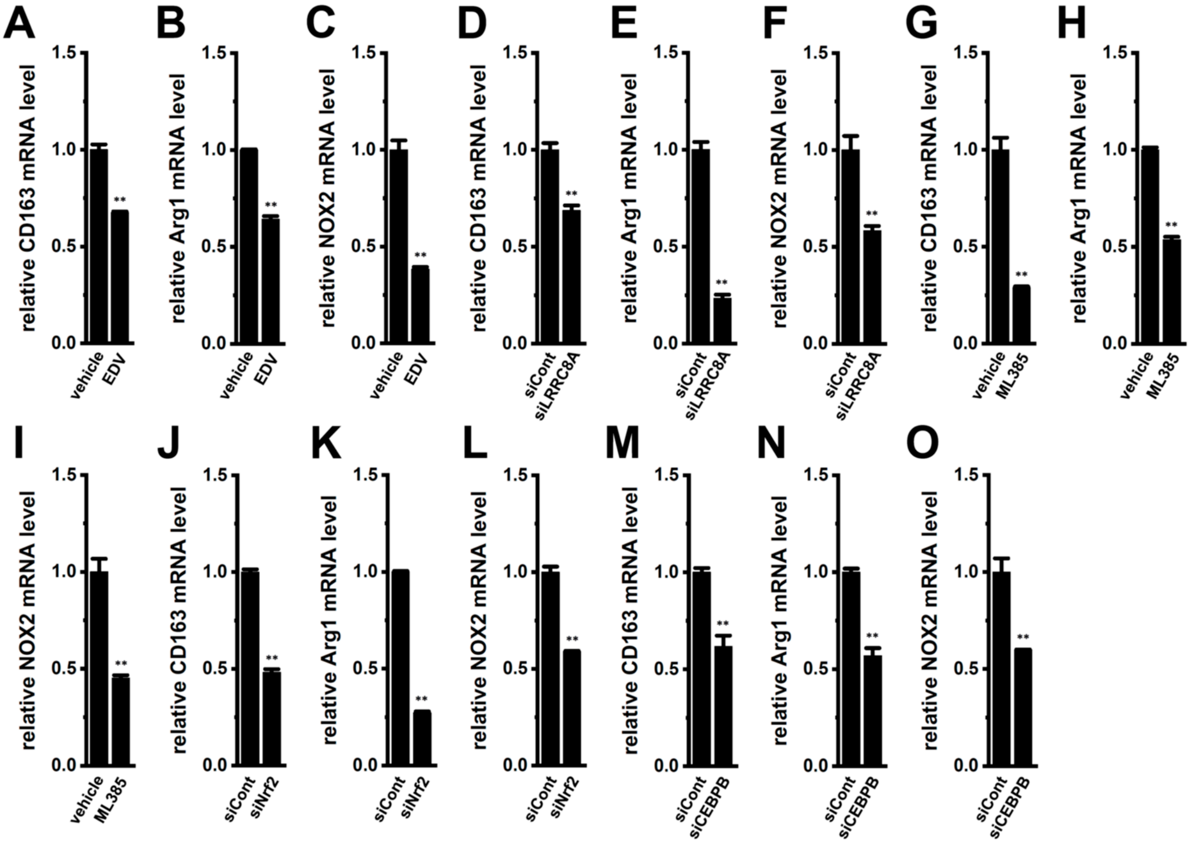

Figure S12. Effects of the pharmacological and/or siRNA-mediated inhibition of LRRC8A, Nrf2, and CEBPB on CD163, Arg1, and NOX2 expression in M$_2$-MACs. A-O: Real-time PCR examination of CD163 (A, D, G, J, M), Arg1 (B, E, H, K, N), and NOX2 (C, F, I, L, O) expression in M$_2$-MACs treated with EDV (10 μM) (A-C) and ML385 (10 μM) (G-I) for 12 hr and transfected with siCont, siLRRC8A (D-F), siNrf2 (J-L), and siCEBPB (M-O). The mRNA level in the vehicle control and siCont group is expressed as 1.0 (*n* = 4 for each). **: *P* <0.01 vs. the vehicle control and siCont.

Target name	Genbank accession number	Primer sequence		Amplicon length (bp)
		Forward (5' to 3')	Reverse (5' to 3')	
IL-8	BC013615	CACTGCGCCAACACAGAAAT	TGAATTCTCAGCCCTCTTCAAAA	120
IL-10	NM_000572	GGCGCTGTCATCGATTTCTT	AGATGTCAAACTCACTCATGGCTTT	120
Nrf2	NM_006164	AACTGGAAAATATAGTAGAA	TGAGTTGTTTTTTCAGTAGG	120
CEBPA	NM_001287435	CCAGGCGGTGGCTTCTC	GGCATTGGAGCGGTGAGTT	121
CEBPB	NM_001285878	GCCCTCGCAGGTCAAGAG	TGCGCACGGCGATGT	107
CEBPD	NM_005195	TCCTGTTGATGCAGCTAAGGTACA	GCATGCTCAGTCTTTTCCTCTTATC	120
CEBPE	NM_001805	CAAGGGCAAGAAGGCAGTGA	TGCTGCGTCTCCAGAATGC	120
CEBPG	NM_001806	ACGCCGAGAGAGGAACAACA	TTTGCTTCCAACCGTTCATTC	120
CEBPZ	NM_001195053	GCAAGAGGTCCTGTCTTCAGATG	TCAGTCAGCCAAGCCAGAGA	120
LRRC8A	NM_001127244	ACCCCAACTCCACCATTCTG	ACCAGTGCAGTCGGTTCTCAT	120
LRRC8B	NM_015350	CAGCTCCATTGGTTTGCAAA	AAAATGCTCGAGCCTGGAACT	120
LRRC8C	NM_032270	AAGGCCTGAAGACAGATTGGA	CCAGGGTATGGATGAGGACTAA	120
LRRC8D	NM_001134479	TGCCCGATGCTGTCTTTGA	GCAGAGGTGGAGCTCTTGGA	119
LRRC8E	NM_025061	TCTTCTCCAAGCTGGCCTTCT	GCACGGAACGGAAGGAGTAC	120
ANO1	NM_018043	TCCACGGAGTCGGGTTTGT	GCCACGGGTCTCATTAATGTG	120
ANO2	NM_020373	CGCAGCCAGAAAACTCACAGT	ACGGGCAGACAGAATAAACCA	128
ANO6	NM_001025356	AGCATGGCAGCCCATCA	GGGACGGAGAAGGACCAGTAG	120
CLCN1	NM_000083	TTGTGTATCTGCATCGGCAAGT	GTGAATGAGGCAATGACAAAGG	121
CLCN2	NM_004366	TGGAACCGGGATGAAGAGACTAT	CACCGAAGCCACTAGCAATACC	121
CLCN3	NM_001243372	GCAGAGGGTCCTGGTTCTTATATC	TCCAGAGCCACAGGCATATG	120
CLCN4	NM_001830	TGCCTGTCTGCCTTCTGGTAT	ACTGGCACCCTCTGACTGATTC	135
CLCN5	NM_001127899	TAGTTGGTCCCAGCTTATCATCAAG	GGCGCAAACACCTTGACAA	129
CLCN6	NM_001286	GGCTCACGTGGAAAGTGC	TCGCCAAGTTCAGCAATCCA	124
CLCN7	NM_001287	AGGATCTTCTTTGCTTCCATGATC	CGAGTCAAACCTTCCGAAGTTG	120
$CLCNK_A/K_B$	NM_004070	TGCCAGCCCTCCTTCTATGAT	CAGTGTGGTGATGCTGTGGTT	132
CLIC1	NM_001287593	CGCAGGTCGAATTGTTCGT	CGGTGGTAACATTGAAGGTGACT	120
CLIC2	NM_001289	TTAGCCCCAGGTACCAATCCT	ACTCAGGTGAGGGTACCTTGGA	120
CLIC3	NM_004669	ACAGACACGCTGCAGATCGA	GATGAACGCGGAGAACTTGTG	130
CLIC4	NM_013943	AGTGATGGTGAAAGCATAGGAAACT	CAGGTCTGGTGGCTTCCTTTT	120
CLIC5	NM_001114086	TGATGCTGAAGGTTGGAAGAA	AAGAGGCGCTGAGAGAAAGGA	120
CLIC6	NM_001317009	AAAACACGAAGAAGGATGCAAA	CTCGGTGCTGTAGGCATCTATTT	120
VDAC1	NM_003374	GACGGGCAGTCTGGAAACC	CCACGTGCAAGCTGATCTTC	120
VDAC2	NM_001184783	GGTTCAGCTGTCTTTGGTTATGAG	CTGGAAGTCCCCAGTCCTGTAG	120
VDAC3	NM_005662	GGTGGCTTGCTGGCTATCAG	TGCCATCGTTCACATGTGTGT	120
KCC1	NM_005072	CTCTGTACGTGGTCGCTGACA	TCTGGGCGTCACGAAGGT	120
KCC2	NM_001134771	GTCTGGCGGAAGTGCAAGAT	CTCGACCTCCGCAGTGATG	120
KCC3	NM_133647	TTTGCTTTGCTTCGATTGGA	AAAGGTGAGGAGGCCAGGAT	120
KCC4	NM_006598	TCAAGGATGCACAGAAGTCCAT	CTCGTAAGACCACGCCTTCAA	120
CFTR	NM_000492	ACTCCAGCATAGATGTGGATAGC	TTTCGAGAGTTGGCCATTCT	126
Ostm1	NM_014028	TCGAACTTTCAACTGTTCAGT	TTTTGCTCTGAGTGAAGA	120
NOX1	NM_00705	ATTCACGGCATTGGTGGAA	GcGCCTACAGTGGGAGTCA	120
NOX2	NM_000397	CGGGACACACATGCCTTTG	CTCATCCCAGCCAGTGAGGTA	120
NOX3	NM_01571	TCGAGGCCAAACCCAAGAC	GAGGGTTCCTTGCCAGAAAAT	120
NOX4	NM_016931	CTGGACCTTTGTGCCTGTACTG	TGACCATTCGGATTTCCATGA	120
NOX5	NM_02450	GGCATCACCCCCTTTGCT	TGGAGCTTCATGTTGTCTTGGA	120
Duox1	NM_017434	GCGGCCTCTGAGCAGTTC	CTCACGGCTCCAGTAGCTGTT	144
Duox2	NM_014080	GGGTCTGCAACAACTACTGGATTC	CAACCACTATGTTGTCCTCCAACT	120
CLCA1	NM+AF8-001285	AAAGATTACCTGCAGCAGCTTCA	CATCCGTCAGCAGCACAATT	120
CLCA2	NM+AF8-006536	TTCCACCTCCTCCCACATTC	CTGCGGCTTGTTGTAGTTGAAG	120
CLCA4	NM_012128	GCCTCCAATCACAGTGAATGC	GCAGTCACATTGGCTCCAAGA	120
TMEM206	NM_001198862	GCCCCAGGTATTGCCTTGT	CTGGGTGGTGCAATTCATGTC	120
HDAC1	NM_004964	GGCTGGCAAAGGCAAGTATTA	ACTAGGCTGGAACATCTCCATTAC	117
HDAC2	NM_001527	GTCAAGGAGGCGGCAAAA	GGGTCATGCGGATTCTATGAG	108
HDAC3	NM_003883	AGAGAGTGGCCGCTACTACTGT	GCACGTGGGTTGGTAGAAGTC	120
HDAC4	NM_006037	GCGTGGGTTTCAACGTCAA	TGATGACACCAGCACCACAT	120
HDAC5	NM_005474	GAGGGAGGCCATGACTTGA	GGCCACTGCGTTGATGTT	120
HDAC6	NM_006044	AATTGGGTGTGTCTCTCTTGCT	AGGCTGACAGGTCGATGTAGCT	121
HDAC7	NM_015401	AGGACACCATGCAGATCATTC	CGTCCCAGTCTACAATGAGGAT	122
HDAC8	AF245664	ACCGTGTCCCTGCACAAATT	CTTGTATGCCATCCTGAATGG	121
HDAC9	NM_058176	CAGTGACACCATTTGGAATGAG	CTCACAACAGCAAACCCATTCT	123
HDAC10	CU013303	ACGCCGGATATCACATTGGTT	TAAGAGGTACAGGAGCTTCCCAAGT	150
HDAC11	NM_024827	AGCGTGTGTACATCATGGATGT	TCCAGGTACTCATCATCCTGTGT	121
SIRT1	NM_012238	ATTTTCCATGGCGCTGAGGTA	CCTCCATGGGTTCTTCTAAACTTG	121
SIRT2	NM_012237	GAGCCTGGTGAAGCCTGATA	CTGCAAGGAGGTACCCATGAC	121
SIRT3	NM_012239	CGGCTCTACACGCAGAACAT	GGAAGGGTCTTTGGCAGACT	121
SIRT4	NM_012240	GAGAAGAAGCTCCCGATTGCA	GTGGTCAGCATGGGTCTATCAA	121
SIRT5	NM_012241	GGTGTACCCAGCAGCCATGTT	ACAGGGTCCCTGGAAATGAAA	121
SIRT6	NM_016539	AGTCTTCCAGTGTGGTGTTCCA	TCTCAAAGGTGGTGTCGAACTT	135
SIRT7	NM_016538	GGGCTGCCCTGAAGCTACAT	GGAGTCGCCAGTGAGAAAATG	121
NSUN2	NM_017755	TGCAGCACCTGGCTCAAAG	AGCAGGTAGCAGCGCTTGTT	120
YBX1	NM_004559	ACCAGGGTGCAGGAGAACAA	CTTCTTCATTGCCGTCCTCTCT	120
WNK1	NM_018979	AGCAGCTGCCACCTTTTCC	CAACCGCAGAAGTCACTGTGA	120
WNK2	NM_001282394	GGACATCTGCTCCGGCTTAG	AATCGAGCACGAGGTTTGCT	120
WNK3	NM_020922	TCTGTCCAATGTGGCTGCAA	AGTGAAGCATCTGAGGACATCTCA	120
WNK4	NM_032387	GATGGTGACCTTCCGATTTGA	TCCCGAATCCGTCTGAGAAA	120
ACTB	NM_001101	AGGCCAACCGCGAGAAGATG	GCCAGAGGCGTACAGGGATA	101

Table S1. List of PCR primers used in this study, related to Section 4.4.

Type	Antibody name	Host species	Working dilution	Company	Product code	Observed MW (approx. kDa)
Primary antibody	LRRC8A	rabbit polyclonal	1:2000 (WB)<br>1:100 (ICC)	Abcepta (Sandiago, CA, USA)	AP19519b	95
	ANO1	rabbit polyclonal	1:500 (WB)<br>1:100 (ICC)	Abcam (Cambridge, UK)	ab84915	115
	Phospho-AKT (Ser473)	rabbit polyclonal	1:500 (WB)	BioLegend (San Diego, CA, USA)	649001	60
	AKT	mouse monoclonal	1:800 (WB)	BioLegend (San Diego, CA, USA)	680302	60
	Phospho-Nrf2 (Ser40)	rabbit monoclonal	1:1500 (WB)<br>1:100 (ICC)	Abclonal (Tokyo, Japan)	AP1133	100
	Nrf2	rabbit polyclonal	1:750 (WB)<br>1:100 (ICC)	Abclonal (Tokyo, Japan)	A21508	100
	Phospho-AMPK (Thr172)	mouse monoclonal	1:2000 (WB)<br>1:100 (ICC)	BioLegend (San Diego, CA, USA)	600651	65
	AMPK	mouse monoclonal	1:2000 (WB)<br>1:100 (ICC)	BioLegend (San Diego, CA, USA)	600551	65
	NOX2	rabbit polyclonal	1:1000 (WB)<br>1:100 (ICC)	ProteinTech (Rosemont, MO, USA)	19013-1-AP	60
	NOX4	rabbit polyclonal	1:100 (ICC)	ProteinTech (Rosemont, MO, USA)	14347-1-AP	
	β-actin (ACTB)	mouse monoclonal	1:15000 (WB)	Sigma-Aldrich (St. Louis, MO, USA)	A1978	45
	β-actin (ACTB)	rabbit polyclonal	1:1000 (WB)	MBL (Nagoya, Japan)	PM053	45
Secondary antibody	HRP-conjugated anti-mouse IgG	goat polyclonal	1:15000 (WB)	ThermoFisher Scientific (Waltham, MA, USA)	31430	
	HRP-conjugated anti-rabbit IgG	goat polyclonal	1:7500 (WB)	ThermoFisher Scientific (Waltham, MA, USA)	AP307P	
	Alexa Fluor 488-labeled anti-rabbit IgG	goat polyclonal	1:1000 (ICC)	Abcam (Cambridge, UK)	ab150077	

WB: western blotting, ICC: immunocytochemistry, MW: molecular weight, HRP: horseradish peroxidase; MBL: Medical & Biological Laboratories

Table S2. List of antibodies used in this study, related to Sections 4.5. and 4.7.
